# Prevalence and risk factors of moderate to severe obstructive sleep apnea syndrome in major depression: a observational and retrospective study on 703 subjects

**DOI:** 10.1186/s12890-017-0522-3

**Published:** 2017-12-04

**Authors:** Matthieu Hein, Jean-Pol Lanquart, Gwenolé Loas, Philippe Hubain, Paul Linkowski

**Affiliations:** Department of Psychiatry and Sleep Laboratory, Erasme Hospital, Université Libre de Bruxelles, ULB, Route de Lennik, 808-1070 Anderlecht Brussels, Belgium

**Keywords:** Major depression, Obstructive apnea syndrome, Prevalence, Risk factors

## Abstract

**Background:**

Several studies have investigated the prevalence and risk factors of depression in subjects with obstructive sleep apnea syndrome. However, few studies have investigated the prevalence and risk factors for obstructive sleep apnea syndrome in major depression. The aim of this study was to examine the prevalence and risk factors of moderate to severe obstructive sleep apnea syndrome in a large sample of individuals with major depression.

**Methods:**

Data from 703 individuals with major depression recruited from the research database of the sleep laboratory of the Erasme Hospital were analysed. An apnea-hypopnea index of ≥15 events per hour was used as cut-off score for moderate to severe obstructive sleep apnea syndrome. Logistic regression analyses were conducted to examine clinical and demographic risk factors of moderate to severe obstructive sleep apnea syndrome in major depression.

**Results:**

The prevalence of moderate to severe obstructive sleep apnea syndrome in major depression is 13.94%. Multivariate logistic regression analysis revealed that male gender, snoring, excessive daytime sleepiness, lower insomnia complaint, presence of metabolic syndrome, age ≥ 50 years, BMI >30 kg/m^2^, ferritin >300 μg/L, CRP >7 mg/L and duration of sleep ≥8 h were significant risk factors of moderate to severe obstructive sleep apnea syndrome in major depression.

**Conclusion:**

Moderate to severe obstructive sleep apnea syndrome is a common pathology in major depression. The identification of these different risk factors advances a new perspective for more effective screening of moderate to severe obstructive sleep apnea syndrome in major depression.

## Background

Obstructive sleep apnea syndrome (OSA) is characterized by repetitive episodes of upper airway obstruction that occur during sleep and is usually associated with a reduction in blood oxygen saturation [1]. The clinical manifestations of OSA include witnessed apneas, snoring, choking/gasping episodes, excessive daytime sleepiness, non-restorative sleep, nocturia, sleep fragmentation/sleep maintenance insomnia, total sleep amount, morning headaches, loss of libido, irritability, and decreased concentration and memory [2]. Some of these symptoms are also present in mental pathologies, such as major depression, which may lead to an under-diagnosis of OSA in these subjects [3]. Both major depression and moderate to severe OSA (apnea-hypopnea index (AHI) ≥15/h) [4] are associated with a higher risk of cardiovascular morbidity and mortality [5, 6], which justifies the need for effective treatment [7].

The co-occurrence of major depression and OSA may have a negative impact on the quality of life and is very frequent [8, 9]. Indeed, in individuals with OSA, the prevalence of depressive affects may reach 63% [10], whereas in individuals with major depression, the prevalence of OAS (AHI ≥5/h) was 36.3% [11]. However, few studies have investigated the prevalence of moderate to severe OSA in major depression. In one example, Ong et al. [12] found a prevalence of 39% of this syndrome in a population of 51 individuals with major depression, whereas in the general population, the prevalence is 1–14% (9–14% of men and 2–7% of women) [13]. Thus, moderate to severe OSA appears to be more common in individuals with major depression than in the general population.

The classical OSA risk factors are age, male gender, body mass index (BMI), snoring, high blood pressure, metabolic syndrome, and sleep duration ≥8 h [14–17]. Although some of these OSA risk factors have been studied in major depression [11, 12, 18, 19], the majority have not been validated for moderate to severe OSA in the particular subpopulation of individuals with major depression.

Regarding alcohol consumption, smoking, benzodiazepines and Z-drugs use, data in the literature are contradictory concerning their potentially promoting effect in obstructive apneas [20–23]. Excessive daytime sleepiness is a common symptom in individuals with OSA [24] and may be measured by the use of the Epworth scale (ESS) [2]. Nevertheless, its use as a predictor of OSA is controversial in the general population [25, 26] and in major depression [18]. Additionally, even though the severity of depression is positively correlated with AHI [27], it does not predict the presence of OSA in major depression [12]. The use of these risk factors is therefore contradictory in the literature. Hence, it would be interesting to study such risk factors with a large sample of individuals with major depression to determine if, in this subpopulation, they are associated with a higher risk of moderate to severe OSA.

Furthermore, in major depression and OSA, there are arguments for the presence of chronic systemic inflammation resulting in higher levels of C-reactive protein (CRP) and ferritin [28–30]. In addition, OSA severity is correlated with the markers of this chronic inflammation [31, 32] that have never been studied as a predictor of OSA in either the general population or those with major depression. Therefore, it would be interesting to investigate if the presence of an inflammatory syndrome is associated with a higher risk of moderate to severe OSA in the subpopulation of major depressed individuals.

Our first objective is to investigate the actual prevalence of moderate to severe OSA in the particular subpopulation of individuals with major depression. Our second objective is to identify in this subpopulation, the specific risk factors of moderate to severe OSA. To achieve these goals, we recruited a large sample of major depressed individuals that we divided into a control group without moderate to severe OSA and a patient group with moderate to severe OSA. The aim of this approach is to enable health professionals who treat those with major depression to reference reliable data concerning this particular problem in this subpopulation and to better identify those at risk of moderate to severe OSA, a diagnosis currently made difficult by the existence of an overlap between symptoms of major depression and OSA.

## Methods

### Population

The 703 individuals with major depression were recruited from the database of the sleep laboratory of Erasme Hospital, which contains data for 3511 individuals who completed sleep laboratory monitoring in the years 2002–2014. In our study, we did not recruit subjects without major depression because our objective is to focus on the subpopulation of those with major depression where the existence of an overlap between the symptoms of major depression and OSA makes the diagnosis of this syndrome more difficult. Physicians specializing in sleep medicine referred these individuals to the sleep laboratory following an ambulatory consultation to evaluate their complaint of poor sleep and their depressive affects.

The inclusion criteria were age ≥ 18 years and the presence of a major depressive episode meeting the diagnostic criteria of the Diagnostic and Statistical Manual of Mental Disorders fourth edition - Text Revision (DSM IV-TR) [33]. The exclusion criteria were presence of a psychiatric disorder other than major depression, presence of uncontrolled heavy somatic disease, presence of chronic pulmonary disease, presence of inflammatory or infectious disease, presence or history of cranial trauma, presence or history of central nervous system injury that could involve respiratory centres in the brain, presence or history of craniofacial or thoracic cavity malformations, presence of pregnancy, presence of OSA already known or course of treatment before sleep laboratory, presence of predominantly central apnea syndrome, presence of narcolepsy or primary hypersomnia, presence of parasomnia, and presence or history of substance abuse.

### Methods

#### Medical and psychiatric evaluation of participants

All subjects upon admission to the sleep laboratory of Erasme Hospital had their medical records reviewed and a complete somatic check-up performed, including a blood test, electrocardiogram, a daytime electroencephalogram, urinalysis, and a chest X-ray (only for those over age 45). These steps allowed for a systematic diagnosis of potential somatic pathologies present in people admitted to our unit.

Metabolic syndrome was diagnosed when three or more of the following criteria were fulfilled: fasting blood glucose ≥100 mg/dl or receiving treatment for diabetes mellitus, blood pressure ≥ 135/85 mmHg or receiving antihypertensive drug treatment, serum triglycerides ≥150 mg/dl, serum HDL-Cholesterol <40 mg/dl or receiving treatment for dyslipidemia, and waist circumference ≥ 94 cm for men or ≥80 cm for women [34, 35].

Patients also benefited on the day of admission from an appointment with a unit psychiatrist who potentially assigned psychiatric diagnoses per the DSM IV-TR criteria [33] to exclude subjects with psychiatric disorders other than major depression.

On admission, patients completed a series of self-questionnaires to assess the severity of their subjective complaints of depression, poor sleep, and excessive daytime sleepiness as follows:

- The presence of depressive symptoms was investigated using the Beck Depression Inventory (BDI reduced to 13 items). This scale consists of 13 items that can be scored from 1 to 3. The final score can vary from 0 to 39. A final score of 0–4 indicates an absence of depression, 5–7 a slight depression, 8–15 a moderate depression, and >16 severe depression [36].
- Daytime sleepiness was investigated using the Epworth scale. This scale consists of eight questions that can be scored from 0 to 3 and assesses sleepiness during different daytime situations. The final score varies from 0 to 24. A final score greater than 10 indicates excessive daytime sleepiness [37].
- The presence of insomnia symptoms was investigated using the Insomnia Severity Index (ISI). This index consists of seven questions that can be scored from 0 to 4. The final score can vary from 0 to 28. A score of 0–7 indicates a lack of insomnia, 8–14 subclinical insomnia,15–21 moderate insomnia. and 22–28 severe insomnia [38].

To avoid missing values, individuals who did not respond fully to these questionnaires were not included in our study.

### Sleep evaluation and study

A psychiatrist of the unit conducted a specific interview focused on sleep on the day of admission to complete an assessment of complaints related to sleep.

Participants stayed in a sleep laboratory for two nights, including a first night of habituation and a night of polysomnography from which the data were collected for analysis. The patients went to bed between 22:00–24:00 and got up between 6:00–8:00, following their usual schedule. During bedtime hours, the subjects were recumbent and the lights were turned off. Daytime naps were not permitted.

The polysomnographic recordings from our unit met the guidelines of the American Academy of Sleep Medicine (AASM) [39]. The applied polysomnography-montage was as follows: two electro-oculogram channels, three electroencephalogram channels (Fz-Ax, Cz-Ax, and Oz-Ax, where Ax was a contralateral mastoid reference), one submental electromyogram channel, electrocardiogram, thermistors to detect the oro-nasal airflow, finger pulse-oximetry, a microphone to record breathing sounds and snoring, piezoelectric sensors and leg movement electrodes. In addition, the applied polysomnography-montage also included strain gauges to measure thoracic and abdominal breathing. Polysomnographic recordings were visually scored by specialized technicians using AASM criteria [40] (inter-judge agreement score of 85%).

Apneas were scored if the decrease in airflow was ≥90% for at least 10 s and hypopneas were scored if the decrease in airflow was ≥30% for at least 10 s with a decrease in oxygen saturation of 3% or followed by a micro-arousal [41]. AHI corresponds to the total number of apneas and hypopneas divided by period of sleep in hours. OSA was considered moderate to severe when AHI was ≥15/h [4].

### Statistical analyses

Statistical analyses were performed using Stata 14. The normal distribution of the data was verified using histograms, boxplots, and quantile-quantile plots, and the equality of variances was checked using the Levene’s test.

We divided our sample of major depressed subjects into a control group without moderate to severe OSA and a patient group with moderate to severe OSA.

Categorical data were described with percentages and numbers, and continuous data were described with means and SD or median and P25-P75. Normally distributed variables were analysed with a *t*-test. A Wilcoxon test or chi [2] test were used on asymmetric distributed or dichotomous variables.

Univariate and multivariate binary logistic regression models were used to study the effects of risk factors on the occurrence of AHI ≥15. Risk factor variables included ESS score (categorical: ≤10, >10), ISI score (categorical: <15, ≥15), BDI score (categorical: <12, ≥12), BMI (categorical: <25 kg/m [2], ≥25 & <30 kg/m [2], ≥30 kg/m^2^), age (categorical: <50 years, ≥50 years), self-reported sleep duration (categorical: <8 h, ≥8 h), ferritin (categorical: ≤300 μg/L, >300 μg/L), CRP (categorical: ≤7 mg/L, >7 mg/L), and as binary variables gender, snoring, metabolic syndrome, benzodiazepines use, Z-drugs use, antidepressants therapy, smoking, and alcohol consumption.

The automatic selection of risk factors in the model was performed by a stepwise backward method with an entry threshold of 0.05 and an exit threshold of 0.1. The adequacy of the model was verified by the Hosmer and Lemeshow test and the specificity of the model by Link Test. The numbers of subjects by risk factors, outliers, and collinearity between risk factors that may cause problems, have also been verified.

A *p*-value of less than 0.05 was considered significant.

## Results

### Demographic data (Table 1)

Male gender, snoring, metabolic syndrome, Z-drugs use and alcohol consumption are more frequent in subjects with AHI ≥15/h. These subjects also present an age/BMI/ESS score greater and BDI/ISI score lower than the subjects with AHI <15/h. Markers of chronic inflammation, such as CRP and ferritin, are higher in moderate to severe OSA. There was no significant difference in benzodiazepines use, antidepressants therapy, smoking, and duration of sleep ≥8 h.

**Table 1:** Sample description (*n* = 703)

Variables		Categories	%	Group AHI < 15/h	Group AHI ≥ 15/h	*P*-value Chi^2^
Gender		Male (*n* = 320)	45.52%	38.68%	87.76%	<0.001
		Female (*n* = 383)	54.48%	61.32%	12.24%	
Snoring		No (*n* = 342)	48.65%	53.22%	20.41%	<0.001
		Yes (*n* = 361)	51.35%	46.78%	79.59%	
Metabolic syndrome		No (*n* = 636)	90.47%	93.72%	70.41%	<0.001
		Yes (*n* = 67)	9.53%	6.28%	29.59%	
Benzodiazepines use		No (*n* = 534)	75.96%	76.03%	75.51%	0.911
		Yes (*n* = 169)	24.04%	23.97%	24.49%	
Z-drugs use		No (*n* = 650)	92.46%	91.57%	97.96%	0.026
		Yes (n = 53)	7.54%	8.43%	2.04%	
Antidepressant therapy		No (*n* = 408)	58.04%	58.51%	55.10%	0.526
		Yes (*n* = 295)	41.96%	41.49%	44.90%	
Smoking		No (*n* = 528)	75.11%	74.21%	80.61%	0.174
		Yes (*n* = 175)	24.89%	25.79%	19.39%	
Alcohol		No (*n* = 548)	77.95%	79.50%	68.37%	0.014
		Yes (*n* = 155)	22.05%	20.50%	31.63%	
	Mean ± SD					t-test
BMI (kg/m^2^)	27.32 ± 6.31			26.69 ± 6.14	31.24 ± 5.94	<0.001
		<25 (*n* = 300)	42.67%	47.60%	12.25%	
		≥25 & <30 (*n* = 191)	27.17%	26.45%	31.63%	
		≥30 (*n* = 212)	30.16%	25.95%	56.12%	
Age (years)	44.96 ± 12.29			43.55 ± 11.95	53.66 ± 10.68	<0.001
		<50 (*n* = 463)	65.86%	70.91%	34.69%	
		≥50 (*n* = 240)	34.14%	29.09%	65.31%	
Sleep duration (hour)	8.26 ± 0.89			8.26 ± 0.88	8.25 ± 0.95	0.917
		<8 (*n* = 394)	56.05%	57.19%	48.98%	
		≥8 (*n* = 309)	43.95%	42.81%	51.02%	
	Median (P25-P75)					Wilcoxon test
Ferritin (μg/L)	102 (48–209)			89 (42–184)	261.5 (134–406)	<0.001
		<300 (*n* = 606)	86.20%	90.74%	58.16%	
		≥300 (*n* = 97)	13.80%	9.26%	41.84%	
CRP (mg/L)	1.6 (0.9–3.5)			1.5 (0.87–3.4)	1.95 (1–3.7)	0.041
		≤7 (*n* = 643)	91.47%	92.30%	86.73%	
		>7 (n = 60)	8.53%	7.7%	13.27%	
BDI	12 (9–16)			12 (10–16)	11 (9–14)	0.025
		<12 (*n* = 331)	47.08%	45.79%	55.10%	
		≥12 (*n* = 372)	52.92%	54.21%	44.90%	
ISI	18 (15–21)			19 (16–22)	15 (12–19)	<0.001
		≥15 (n = 548)	77.95%	81.49%	96.12%	
		<15 (n = 155)	22.05%	18.51%	3.88%	
Epworth	11 (7–14)			10 (6–14)	11.5 (9–15)	0.001
		≤10 (*n* = 346)	49.22%	51.24%	36.73%	
		>10 (*n* = 357)	50.78%	48.76%	63.27%	
AHI	2 (1–7)	<15/h (*n* = 605)	86.06%			
		≥15/h (n = 98)	13.94%			

*BMI* Body Mass Index, *ISI* Insomnia Severity Index, *BDI* Beck Depression Inventory, *AHI* Apnea-Hypopnea Index

### Prevalence of moderate to severe OSA in major depression (Table 1)

The prevalence of moderate to severe OSA in our sample of 703 individuals with major depression is 13.94% (*n* = 98).

### Univariate analysis (Table 2)

Male gender, snoring, ESS score > 10, ISI score < 15, metabolic syndrome, Z-drugs use, alcohol consumption, age ≥ 50 years, BMI ≥25 & <30 kg/m^2^, BMI >30 kg/m^2^, and ferritin >300 μg/L were associated with an increased risk of moderate to severe OSA in major depression.

**Table 2:** Univariate analysis (n = 703)

Variables	% AHI <15/h	% AHI ≥15/h	OR (IC 95%)	*P*-value
Gender				<0.001
Male	73.12%	26.88%	11.36 (6.08 to 21.24)	
Female	96.87%	3.13%	1	
Snoring				<0.001
Yes	78.39%	21.61%	4.44 (2.65 to 7.44)	
No	94.15%	5.85%	1	
Epworth				0.008
≤ 10	89.60%	10.40%	1	
> 10	82.63%	17.37%	1.81 (1.16 to 2.81)	
ISI				<0.001
≥ 15	89.96%	10.04%	1	
< 15	72.26%	27.74%	3.44 (2.20 to 5.39)	
BDI				0.088
< 12	83.69%	16.31%	1	
≥ 12	88.17%	11.83%	0.69 (0.45 to 1.06)	
Metabolic syndrome				<0.001
Yes	56.72%	43.28%	6.27 (3.64 to 10.81)	
No	89.15%	10.85%	1	
Benzodiazepines use				0.911
Yes	85.80%	14.20%	1.03 (0.63 to 1.69)	
No	86.14%	13.86%	1	
Z-drugs use				0.042
Yes	96.23%	3.77%	0.23 (0.05 to 0.95)	
No	85.23%	14.77%	1	
Antidepressant therapy				0.526
Yes	85.08%	14.92%	1.15 (0.75 to 1.77)	
No	86.76%	13.24%	1	
Smoking				0.176
Yes	89.14%	10.86%	0.69 (0.41 to 1.18)	
No	85.04%	14.96%	1	
Alcohol				0.015
Yes	80%	20%	1.79 (1.12 to 2.87)	
No	87.77%	12.23%	1	
BMI (kg/m^2^)				<0.001
< 25	96%	4%	1	
≥ 25 & <30	83.77%	16.23%	4.65 (2.32 to 9.31)	
≥ 30	74.06%	25.94%	8.41 (4.37 to 16.17)	
Age (years)				<0.001
< 50	92.66%	7.34%	1	
≥ 50	73.33%	26.67%	4.59 (2.92 to 7.21)	
Sleep duration (hour)				0.130
< 8	87.82%	12.18%	1	
≥ 8	83.82%	16.18%	1.39 (0.91 to 2.13)	
Ferritin (μg/L)				<0.001
< 300	90.59%	9.41%	1	
≥ 300	57.73%	42.27%	7.05 (4.34 to 11.47)	
CRP (mg/L)				0.074
≤ 7	86.78%	13.22%	1	
> 7	78.33%	21.67%	1.82 (0.94 to 3.50)	

*BMI* Body Mass Index, *ISI* Insomnia Severity Index, *BDI* Beck Depression Inventory, *AHI* Apnea-Hypopnea Index

### Multivariate analysis (Table 3)

In major depression, risk factors associated significantly with an increased risk of moderate to severe OSA and obtained by the method of automatic selection (stepwise backward) were male gender, snoring, ESS score > 10, ISI score < 15, metabolic syndrome, age ≥ 50 years, BMI >30 kg/m^2^, ferritin >300 μg/L, CRP >7 mg/L, and duration of sleep ≥8 h.

**Table 3:** Summary of logistic regression for screening variables predicting AHI ≥15/h in major depression (n = 703)

Variables	Adjusted OR (IC 95%)	*P*-value
Gender		<0.001
Male	10.71 (5.02 to 22.86)	
Female	1	
Snoring		0.007
Yes	2.36 (1.27 to 4.39)	
No	1	
Epworth		0.002
≤ 10	1	
> 10	2.45 (1.39 to 4.35)	
Ferritin (μg/L)		0.009
< 300	1	
≥ 300	2.27 (1.22 to 4.20)	
CRP (mg/L)		0.039
≤ 7	1	
> 7	2.76 (1.05 to 7.23)	
BMI (kg/m^2^)		0.019
< 25	1	
≥ 25 & <30	2.06 (0.93 to 4.59)	
≥ 30	2.62 (1.20 to 5.73)	
Age (years)		<0.001
< 50	1	
≥ 50	3.42 (1.94 to 6.02)	
ISI		<0.001
< 15	3.51 (1.96 to 6.29)	
≥ 15	1	
Metabolic syndrome		0.015
Yes	2.45 (1.19 to 5.02)	
No	1	
Sleep duration (hour)		0.039
< 8	1	
≥ 8	1.77 (1.03 to 3.05)	

Not included in the model because not significant: BDI, benzodiazepines, Z-drugs, antidepressants, smoking and alcohol

Adequacy of model: Hosmer-Lemeshow chi2 (*p* = 0.863)

Specificity of model: Linktest (linear component *p* < 0.001 and nonlinear component *p* = 0.638)

*BMI* Body Mass Index, *ISI* Insomnia Severity Index, *BDI* Beck Depression Inventory, *AHI* Apnea-Hypopnea Index

## Discussion

In our sample of individuals with major depression, we demonstrated a prevalence of moderate to severe OSA of 13.94%, which highlights the importance of this problem to the healthcare professionals treating this particular subpopulation of patients. This prevalence is similar to that of the general population [13], but less than that of 39% of the study of Ong et al. [12] However, in this study, the sample was relatively small and to be included, these individuals had to present with insomnia meeting the diagnostic criteria of DSM-IV-TR [33]. This diagnostic criteria included difficulty initiating or maintaining sleep, non-restorative sleep, clinically significant distress, or impairment in social, occupational, or other important areas of functioning; all of which are also symptoms of OSA [2] and may result in greater recruitment of patients with major depression and moderate to severe OSA and could explain the difference in prevalence within our study. Moreover, although the prevalence of moderate to severe OSA in major depression appears to be similar to that of the general population as indicated by our results, the existence of an overlap between the symptoms of major depression and OSA [42] as well as non-compliance with medical treatment in individuals with major depression [43] may lead to the under-diagnosis of moderate to severe OSA in major depression [3]. However, moderate to severe OSA is associated with increased cardiovascular morbidity and mortality [44], which justifies the implementation of effective treatment [45]. Therefore, in individuals with major depression, it is important to identify the specific risk factors for moderate to severe OSA to enhance the detection and management of this syndrome and reduce cardiovascular complications for these individuals.

As in the general population [14], we found that male gender, age ≥ 50 years, and BMI ≥30 kg/m^2^ are risk factors for moderate to severe OSA in major depression, which seems to confirm the results of Ong et al. [12] Furthermore, although snoring is a risk factor for mild to severe OSA in major depression [18], it has not been studied specifically for moderate to severe OSA. Nevertheless, in our study, we have demonstrated that similar to the general population [14], snoring is also a risk factor for moderate to severe OSA in individuals with major depression. We therefore confirmed with a large sample that the classical risk factors for moderate to severe OSA in the general population are applicable to the subpopulation of individuals with major depression, which seems to confirm the results of preliminary studies involving smaller samples of individuals with major depression [12, 18].

In the general population, there is a special relationship between OSA and metabolic syndrome. Indeed, subjects with a metabolic syndrome have a higher risk of severe OSA [17], and individuals with moderate to severe OSA have a higher risk of metabolic syndrome [46, 47]. In addition, the prevalence of metabolic syndrome increases with the severity of OSA [48]. However, in major depression, no studies have investigated the relationship between OSA and metabolic syndrome, which are two syndromes that frequently present in individuals with major depression [11, 49]. Another risk factor for moderate to severe OSA found in the general population, but not studied in major depression, is sleep duration ≥8 h [16]. However, in our study, we demonstrated that as in the general population, metabolic syndrome and duration of sleep ≥8 h are also risk factors for moderate to severe OSA in the particular subpopulation of individuals with major depression.

Despite two meta-analyses [25, 26], data in the literature on the use of excessive daytime sleepiness measured by ESS as a predictor of moderate to severe OSA in the general population are contradictory. Yet, in mental pathologies, including major depression, the use of ESS as a risk factor for moderate to severe OSA seems to not be recommended, as demonstrated in the study of Nikolakaros et al. [18] However, in this study, the sample size was small and it did not consist solely of individuals with major depression. In OSA, there is a particular relationship between daytime sleepiness measured by ESS and major depression. Indeed, in subjects with OSA, the presence of excessive daytime sleepiness is associated with a greater risk of depression [50], whereas depressive symptoms contribute significantly to excessive daytime sleepiness [51]. These elements allow better understanding why we have shown that excessive daytime sleepiness is a risk factor of moderate to severe OSA in major depression. Although some studies show a positive correlation between AHI and the severity of depression [27, 52], we have demonstrated a finding similar to Ong et al. [10] where subjects with an AHI ≥15/h had a lower self-reported severity of depression than subjects with AHI <15/h, and that the self-reported severity of depression is not a risk factor for moderate to severe OSA in major depression. In addition, Bjorvatn et al. [53] have shown that the prevalence of insomnia complaints decreased when the severity of OSA increased, which enhances the understanding of our results. Indeed, in our study, we have shown that individuals with major depression and lower self-reported complaints of insomnia had a greater risk of moderate to severe OSA. Therefore, in the subpopulation of those with major depression, excessive daytime sleepiness and lower insomnia complaints are risk factors for moderate to severe OSA, unlike the self-reported severity of depression.

In OSA and major depression, there are arguments in favour of chronic inflammation, which may be correlated with the severity of OSA [31, 32] and which may result in higher plasma levels of CRP and ferritin [54–56]. Despite the special relationship between chronic inflammation and depression/OSA, plasma CRP and ferritin levels have never been studied as a risk factor for OSA in the general population or individuals with major depression. However, in our study, we found that the presence of chronic inflammation in a subpopulation of individuals with major depression was a risk factor for moderate to severe OSA, which advances new perspectives in understanding the relationship between OSA and major depression.

Antidepressants may partially improve OSA by suppressing REM sleep and increasing upper airway tone [57]. However, in our study, we demonstrated that antidepressants are not a risk or protector factor for moderate to severe OSA in individuals with major depression. This can be explained by the fact we did not distinguish between the different classes of antidepressants, which may possibly mask the protective or deleterious effect of certain molecules on respiration. We found that benzodiazepines and Z-drugs are not risk factors for moderate to severe OSA, which seems to confirm the results of the meta-analysis of Mason et al. [23] However, we excluded subjects with dependence and therefore an overconsumption of these molecules. Benzodiazepines and Z-drugs are generally safe at a low dose for nocturnal breathing, but at high doses, they may cause or aggravate sleep apnea in some more fragile patients [57]. Thus, taking benzodiazepines and Z-drugs at a recommended dose, especially for the subpopulation of individuals with major depression, is not a risk factor for moderate to severe OSA.

The role of smoking in the occurrence of obstructive apnea is controversial in the literature [58, 59]. It would appear that nicotine would decrease the resistance of the upper airways with a consequent reduction of the risk of OSA, whereas in case of withdrawal, this resistance would become more important and would cause a greater risk of OSA [60]. Nevertheless, a protective effect of smoking for OSA has yet to be investigated. In our study, we found that smoking is not a risk factor for moderate to severe OSA in the subpopulation of major depressed subjects. This may be explained by the fact that we included only active smokers who did not have nicotine withdrawal during their sleep laboratory. Further, in the literature, alcohol is a recognized risk factor for OSA. In fact, it induces a decrease in the tone of the upper airway muscles, which may increase the frequency and the severity of obstructive apnea in subjects with OSA, especially during the first hours of sleep [61]. Similarly, we demonstrated that alcohol is not a risk factor for moderate to severe OSA in the subpopulation of individuals with major depression. This difference from the literature can be explained by the fact that none of the subjects included in our study had alcohol dependence and thus could stop their habitual consumption of alcohol during the sleep laboratory without consequence and avoid its deleterious effects on nocturnal breathing.

In the future, prospective studies should be conducted with the subpopulation of individuals with major depression to validate the risk factors of moderate to severe OSA highlighted in our study. In addition, it would be useful to develop a score from these risk factors to better identify those at risk of moderate to severe OSA.

### Limitations

The results obtained in our study come from retrospective data that, even if they have been encoded in a systematic manner, cannot be verified directly with the subject in most cases, which means that our results need to be replicated in prospective studies. Further, we used an automatic selection of risk factors by a stepwise backward method, which presents some limitations that can be consulted on http://www.stata.com/support/faqs/statistics/stepwise-regression-problems. Moreover, we focused only on moderate to severe OSA, which means that our results cannot be generalized to other breathing disorders during sleep, such as central apnea syndrome. As we have included only patients with major depression and without other psychiatric comorbidities, our results are not generalizable to all individuals with major depression or other psychiatric disorders.

## Conclusion

We demonstrated in a large sample of individuals with major depression that the prevalence of moderate to severe OSA was 13.94%, and that the classical risk factors for moderate to severe OSA (male gender, age ≥ 50 years, BMI ≥30 kg/m^2^, and snoring) were applicable this particular subpopulation. We also found that the presence of metabolic syndrome, sleep duration ≥8 h, excessive daytime sleepiness, lower insomnia complaints, and markers of chronic inflammation (CRP and ferritin) were also risk factors for this syndrome in the subpopulation of individuals with major depression, unlike self-reported severity of depression, antidepressant therapy, smoking, alcohol consumption, or benzodiazepines and Z-drugs use.

## Highlights

- The prevalence of moderate to severe OSA in major depression is 13.94%.
- Male gender, age ≥ 50 years, BMI ≥30 kg/m^2^, snoring, presence of metabolic syndrome, sleep duration ≥8 h, excessive daytime sleepiness, lower insomnia complaints and markers of chronic inflammation (CRP and ferritin) were risk factors for moderate to severe OAS in major depression.
- These risk factors open up a new perspective for more effective screening of moderate to severe OSA in major depression.

## Abbreviations

AHI: Apnea-hypopnea index
BDI: Beck depression inventory
BMI: Body mass index
CRP: C-reactive protein
DSM IV-TR: Diagnostic and Statistical Manual of Mental Disorders fourth edition - Text Revision
ESS: Epworth scale
ISI: Insomnia severity index
OSA: Obstructive sleep apnea syndrome
